# Simultaneous Detection of Ca^2+^ and Diacylglycerol Signaling in Living Cells

**DOI:** 10.1371/journal.pone.0042791

**Published:** 2012-08-17

**Authors:** Paul Tewson, Mara Westenberg, Yongxin Zhao, Robert E. Campbell, Anne Marie Quinn, Thomas E. Hughes

**Affiliations:** 1 Montana Molecular, Bozeman, Montana, United States of America; 2 Department of Cell Biology and Neuroscience, Montana State University, Bozeman, Montana, United States of America; 3 Department of Chemistry, University of Alberta, Edmonton, Alberta, Canada; Indiana University School of Medicine, United States of America

## Abstract

Phospholipase C produces two second messengers - diacylglycerol (DAG), which remains in the membrane, and inositol triphosphate (IP_3_), which triggers the release of calcium ions (Ca^2+^) from intracellular stores. Genetically encoded sensors based on a single circularly permuted fluorescent protein (FP) are robust tools for studying intracellular Ca^2+^ dynamics. We have developed a robust sensor for DAG based on a circularly permuted green FP that can be co-imaged with the red fluorescent Ca^2+^ sensor R-GECO for simultaneous measurement of both second messengers.

## Introduction

G protein couple receptors (GPCR) activate heterotrimeric G proteins that in turn interact with many different effectors to alter the levels of intracellular second messengers such as cyclic nucleotides, intracellular Ca^2+^, DAG, and IP_3_. A particular GPCR, acting through one type of heterotrimeric G protein, can alter the activity of multiple effectors and second messengers such that the signal that is generated within the cell involves a complex pattern of second messenger signaling coordinated in space and time. To understand this pattern of activity, and unambiguously determine which G-protein pathway causes it, new multiplex sensor systems are needed that can simultaneously measure multiple second messengers.

Many cell surface receptors couple to the heterotrimeric G protein Gq, which in turn activates Phospholipase C (PLC). PLC produces two different second messengers, DAG and IP_3_, and ultimately the IP_3_ causes an increase in intracellular Ca^2+^. It is this coordinated increase of both DAG and cytosolic Ca^2+^ that triggers the activation of conventional isoforms of protein kinase C (cPKC) which in turn phosphorylate many different protein targets. To date, the most robust fluorescent sensors for this pathway detect Ca^2+^, but a rise in Ca^2+^ is an ambiguous signal: there are other signaling pathways that cause increases in intracellular Ca^2+^. To unambiguously resolve PLC pathway activation, and to better understand the kinetics of these coordinated, parallel signaling processes in health and disease [1], we developed a robust sensor system for the simultaneous detection of DAG and Ca^2+^.

Several genetically encoded, fluorescent DAG sensors have been described. The simplest of these are composed of a green FP fused to the C1 domain of a conventional PKC [2]–[4]. This C1 domain translocates to the membrane and binds DAG when it is generated, so the physical translocation of the fluorescent protein, the membrane localization of the fluorescence, becomes the measurement of DAG signaling. The limitation of this approach is that it is not very quantitative, and it requires high resolution optical imaging, or TIRF illumination, to detect the intracellular translocation event. More recently, a sensor was created in which Förster resonance energy transfer (FRET) efficiency between two different FPs changes in response to elevated levels of DAG [5]–[7]. Like the translocation sensors, this probe is useful in high resolution microscopy, but the changes in emission ratio of the donor and acceptor FPs upon sensor activation are relatively small.

Currently, the FP-based Ca^2+^ sensors GCaMP3 [8], G-GECO, and R-GECO [9] are the most robust class of genetically encoded fluorescent tools. These are the result of many years of of optimization in multiple research groups. Members of this class of sensors are constructed from a single circularly permuted fluorescent protein with calcium-dependent binding partners attached to the new termini. The crystal structure of one of these single FP-based sensors revealed that the binding partners cause a change in fluorescence intensity by opening and closing a hole in the protein β-barrel in close proximity to the chromophore [8]. Mutations that better occlude the hole in the Ca^2+^ bound state produced an even better Ca^2+^ sensor GCaMP3, which was in turn improved upon to create G-GECO and eventually, R-GECO.

**Figure 1:**
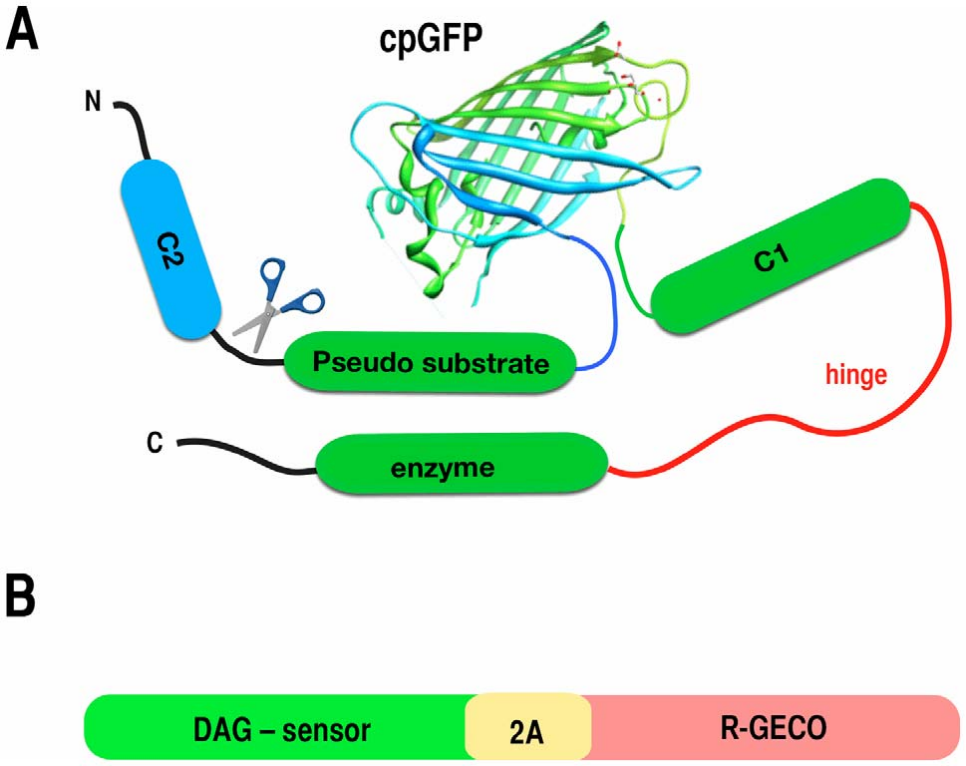
Sensor design. (**A**) To create potential DAG sensors, we inserted the cpGFP from G-GECO into the region interconnecting the pseudo substrate and the C1 domain, or in the hinge that connects the C1 domain with the enzyme (red). Some constructs were created with the entire PKCδ, in others the C2 domain was removed. (**B**) To pair the Upward or Downward DAG sensors with R-GECO, we connected the two coding regions, in frame, with an intervening 2A peptide sequence of 17 amino acids.

To determine whether a conceptually analogous sensor could be made for DAG, we created a variety of fusions that placed the circularly permuted green FP from G-GECO1 between the pseudo substrate domain and the C1 domain, or the hinge region, of the PKC isoform PKCδ (Figure 1). The C2 domain of PKCδ is not responsive to Ca^2+^ [10], and the C1 domain has a high affinity for DAG [11]. Reasoning that DAG binding to the C1 domain separates the pseudo substrate from the enzyme, we positioned the circularly permuted FP in portions of the PKCδ that could conceivably undergo large conformational alterations following DAG binding.

## Methods

### Plasmid/Sensor Construction

Small changes in the exact fusion sites and linker composition can make large differences in the response properties of sensors based on circularly permuted FPs [12], so we created an initial test set of 64 fusion proteins (table 1) in which we systematically adjusted the position of the fusion site and/or removed the C2 domain. Sixty four different prototypes of a DAG sensor were created by fusing a circularly permuted green FP from G-GECO to 30 different positions within the novel PKCδ isoform. PCR amplification was used to generate fragments of PKCδ the coding region for the cpEGFP of G-GECO. Different combinations of PKC fragments were then paired with the cpEGFP amplicon and cloned into a modified version of the mammalian expression vector pcDNA3.1 using the In-Fusion Cloning system (Clontech Laboratories Inc, Mountain View, CA). The pcDNA3.1 vector was obtained from Life Technologies (Grand Island, NY). Thirty two of the prototypes involved inserting the cpEGFP into the full length PKCδ, an additional 32 constructs were created in which the N-terminal region of PKCδ containing the C2 domain was deleted.

**Table 1:** Summary of constructs created and tested.

Sensor	FP position	Truncation site	Deletion	Sensor	FP position	Truncation site	Deletion
PcpG1	C280			PcpG16 2B	D217	L122	
PcpG2	I282			PcpG17 2A	N158	L91	
PcpG3	L286			Upward DAG	N158	L122	
PcpG4	A290			PcpG17 2C	N158	L106	
PcpG5	Q296			PcpG17 2D	N158	Q129	
PcpG6	S302			PcpG17 2A	N158	K138	
PcpG7	E308			PcpG18 2B	K157	L122	
PcpG8	Y313			PcpG19 2B	I156	L122	
PcpG9	T320			PcpG20 2B	Y155	L122	
PcpG10	E325			PcpG21 2B	H154	L122	
PcpG11	G332			PcpG22 2B	I153	L122	
PcpG12	I337			Upward DAG	K152	L122	
PcpG13	K343			PcpG24 2B	H159	L122	
PcpG14	N348			PcpG25 2B	E160	L122	
PcpG15	Y448			PcpG26 2B	F161	L122	
PcpG16	D217			PcpG27 2B	I162	L122	
PcpG17	N158			PcpG28 2B	A163	L122	
PcpG1 2B	C280	L122		PcpG29 2B	T164	L122	
PcpG2 2B	I282	L122		PcpG30 2B	E134	L122	
PcpG3 2B	L286	L122		PcpG1-2	C280		G281-I282
PcpG4 2B	A290	L122		PcpG1-3	C280		G281-L286
PcpG5 2B	Q296	L122		PcpG1-4	C280		G281-A290
PcpG6 2B	S302	L122		PcpG1-5	C280		G281-Q296
PcpG7 2B	E308	L122		PcpG1-6	C280		G281-S302
PcpG8 2B	Y313	L122		PcpG1-7	C280		G281-E308
PcpG9 2B	T320	L122		PcpG1-8	C280		G281-Y313
PcpG10 2B	E325	L122		PcpG1-9	C280		G281-T320
PcpG11 2B	G332	L122		PcpG1-10	C280		G281-E325
PcpG12 2B	I337	L122		PcpG1-11	C280		G281-G332
PcpG13 2B	K343	L122		PcpG1-12	C280		G281-I337
PcpG14 2B	N348	L122		PcpG1-13	C280		G281-K343
PcpG15 2B	Y448	L122		PcpG1-14	C280		G281-N348

The sequence encoding the circularly permuted fluorescent protein was inserted into the PKCδcoding region such that fusions occurred just following the amino acid in PKCδ listed. The N-terminus of PKCδ was truncated in some constructs, with the translation start beginning just before the amino acid listed.

### Cell Culture and Transfection

Cells were cultured in EMEM supplemented with 10% fetal bovine serum and Penicillin-Streptomycin at 37°C in 5% CO_2_. HEK 293 cells and Eagle’s Minimum Essential Medium (EMEM) were purchased from ATCC (Manassas, VA). Prior to cell seeding, 96-well glass-bottom plates were coated with Poly-D-Lysine. Cells were seeded on the plates, transfected using Lipofectamine 2000 Transfection Reagent according to the manafacturer’s protocol, and incubated for 48 hours at 37°C in 5% CO_2_. 60 ng of sensor DNA was co-transfected with 40 ng of human M1 muscarinic receptor per well. Pen-Strep liquid and Lipofectamine 2000 were obtained from Life Technologies (Grand Island, NY). Poly-D-Lysine was purchased from Fisher Scientific (Pittsburg, PA).

### Cell Imaging

Prior to fluorescence imaging, EMEM culture medium was replaced with 1X DPBS. Experiments were performed on a Zeiss Axiovert S100TV inverted microscope fitted with computer controlled excitation/emission filter wheels, shutters, and a Qimaging Retiga Exi ccd camera (Surrey, BC Canada). Cells were imaged live at 25°C using the 10X objective lens. 480±20 nm excitation and and 535±25 nm emission filters were used resolve the green fluorescence from the DAG sensors, and 572±20 nm and 630±30 nm filters were used to collect the R-GECO signal. Cells were analyzed for increases or decreases in fluorescence intensity upon addition of Carbachol, PDBU, or Ionomycin. To analyze the image stacks, background fluorescence was defined as a region of the image that contained no cells. The average value of this region was subtracted frame by frame from the measurements of the mean pixel values of the fluorescent cells. Cellular fluorescence data was plotted and analyzed with IGOR software (Wavemetrics, Oswego Ore.).

### Materials

Phorbol 12, 13-dibutyrate (PDBU), Carbachol, and Ionomycin were purchased from Sigma-Aldrich Corp (St. Louis, MO). Dulbecco’s PBS/Modified was purchased from Fisher Scientific (Pittsburg, PA).

## Results

**Figure 2:**
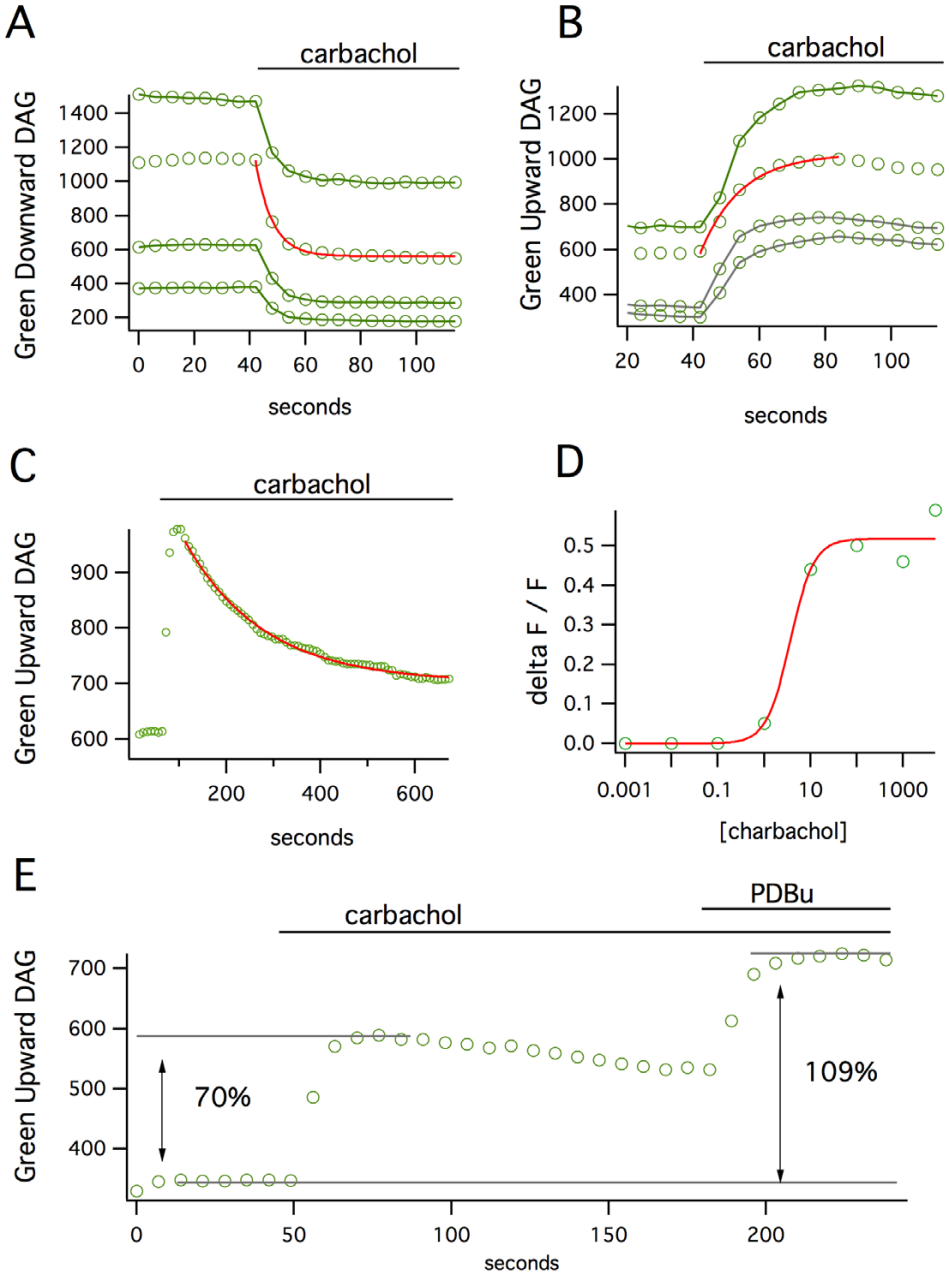
The responses of Green Downward DAG and Upward DAG sensors. (**A**) Carbachol stimulation of the M1 receptor on cells expressing the Downward DAG sensor produces a 40% loss in fluorescence that occurs over ∼15 seconds (mean fluorescence over time of 4 cells). (**B**) The Upward DAG sensor shows a fluorescence increase of 45% over a similar time scale. (**C**) The signals generated by either sensor return to baseline quite slowly. (**D**) The apparent EC_50_ for carbacol-stimulated Upward DAG response is 3.5 uM. (**E**) The carbachol stimulation does not appear to activate all of the sensor pool in the cell since direct activation of the sensors with a subsequent application of PDBu produces an additional increase in fluorescence.

To test the functionality of the 64 fusion proteins, we co-expressed each construct with the M1 acetylcholine receptor, which couples to the Gq signaling pathway, in HEK 293 cells. Application of the agonist carbachol produced no change in fluorescence for most of the constructs, though many of the fusion proteins did translocate to the plasma membrane in response to activation. Of the 10 sensors that did produce a significant change in fluorescence, one sensor produced a remarkable 40% decrease in fluorescence (Green Downward DAG, Figure 2A). A different sensor, in which the FP insertion site was just 6 amino acids away from the first, produced a 45% increase in fluorescence (Green Upward DAG, Figure 2B). These changes were easily detected in time-lapse imaging and occurred in all transfected cells with remarkably little cell to cell variability. The increase or decrease of the signal produced by the Upward or Downward DAG, respectively, was reasonably fit by a single exponential function with a time constant of 6 to 11 seconds. The signals then returned to baseline quite slowly (τ – 170 seconds, Figure 2C).

**Figure 3:**
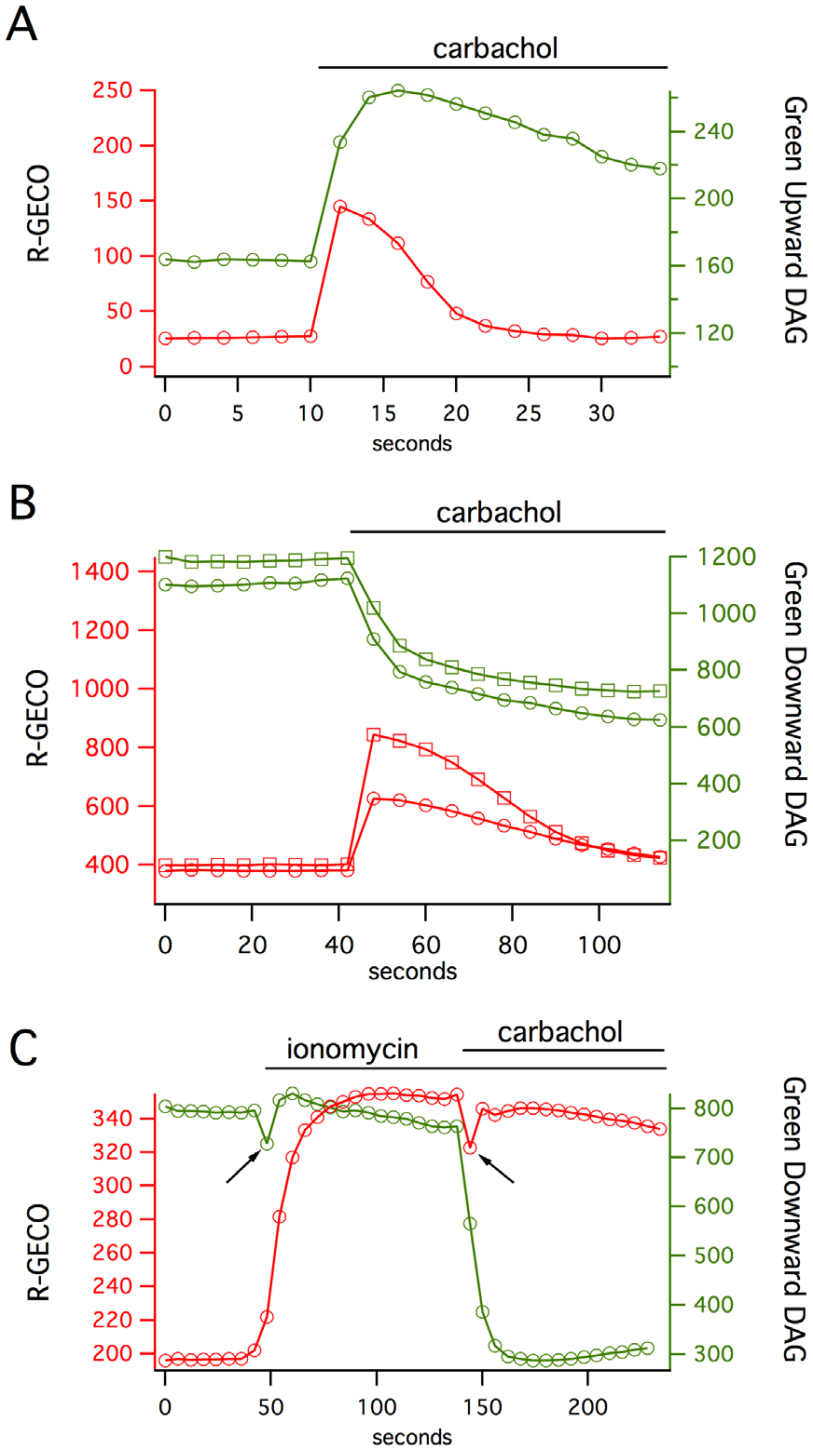
Pairing the Green Upward and Downward DAG sensors with R-GECO makes it possible to simultaneously measure DAG and Ca^2+^ signaling in single cells. (**A**) The Green Upward DAG sensor response is considerably slower than the red Ca^2+^ response in response to carbachol stimulation of the M1 receptor. (**B**) Similar kinetics occur with the Downward DAG sensor. (**C**) The two sensors can be activated independently: ionomycin, which should raise intracellular Ca^2+^ without affecting DAG levels produces a change in R-GECO but not Downward DAG, while the subsequent addition of PDBu activates Downward DAG (arrows indicate stimulus artifact).

Both the Upward and Downward DAG sensors showed robust changes in fluorescence that are an order of magnitude larger than the previously reported, FRET-based DAG sensors. We suspected that our measurements of the maximal sensor responses might be an underestimate. In transient expression it is possible to produce high concentrations of the protein-based sensor than the analyte itself [13]. To test whether this might be occurring, cells were first stimulated with carbachol and then the phorbol ester PDBu was added to directly activate any remaining sensors within the cell (Figure 2D). This produced an additional doubling of the change in intensity, indicating that not all of the sensors in a given cell were activated by the carbachol, and that larger changes in fluorescence might be seen at lower intracellular concentrations of sensor, such as in the context of stable cell lines or transgenic animals.

One advantage of sensors constructed with single FPs is that they use less of the visible spectrum than FRET-based systems. This means that different sensors of different colors can be combined to monitor multiple signaling pathways simultaneously. To multiplex the expression of the DAG sensor with a Ca^2+^ sensor, we fused the coding regions of Green Upward or Green Downward DAG to a cotranslational self-cleaving 2A [14] peptide followed by R-GECO1 [9] (Figure 1B) to produce stoichiometrically balanced proportions of the two sensors. R-GECO1 is a red fluorescent Ca^2+^ sensor based on a circularly permuted red fluorescent protein mApple [15] with excitation and emission properties that are easily distinguished from the green fluorescent DAG sensors.

In cells transiently expressing this dual sensor system, stimulation of the M1 receptor produces a fast rise in intracellular Ca^2+^, as detected by changes in the red fluorescence channel, and a much slower rise in DAG, as detected in the green fluorescence channel (Figure 3). The Ca^2+^ returns to baseline in ∼20 seconds, while the DAG levels remain high for 200–300 seconds. This occurs for either the Downward or Upward DAG sensors paired with R-GECO1. To test for the independence of the signals being detected by these sensors, we increased intracellular Ca^2+^ by applying ionomycin. This triggers a robust R-GECO1 response and no detectable change in the DAG sensor, which was subsequently activated by the addition of PDBu (Figure 3C).

## Discussion

The development of these DAG sensors provides a new avenue for obtaining insights into PLC signaling. Measuring DAG and Ca^2+^ signaling in single cells reveals that PLC signaling appears to operate in two different time zones. Following stimulation, both Ca^2+^ and DAG are elevated for about 10 seconds. This time zone should be when conventional protein kinase C (cPKC) isoforms are active since they require the coordinated binding of both the C1 and C2 domains. The novel PKC isoforms (nPKC), however, should be active over a much longer time zone since elevated DAG levels are sufficient to activate the high affinity C1 domains of the nPKCs [4]. These differences in the kinetics of the signaling pathway responses have been seen with translocation-based sensors in the past [2], but they become more compelling when they can be measured at the same time in the same cell. One can imagine how this enables PLC signaling to affect different targets in different time scales.

To fully understand cell signaling, we will need probes to measure the dynamics of each step in the pathway. Protein-based sensors have made it possible to use protein domains that are exquisitely tuned to detect second messengers in physiological ranges of concentration. Recent advances in the creation of Ca^2+^ sensors, and cGMP [16], have shown that fusions with circularly permuted FPs can produce robust sensors that far exceed FRET-based probes. Here we show that this design is extensible and valuable for robust detection of the crucial second messenger DAG.
