# Correction: Simultaneous Detection of Ca^2+^ and Diacylglycerol Signaling in Living Cells

**DOI:** 10.1371/annotation/108decc5-1a4a-4fa7-a867-d6cfaea8f471

**Published:** 2014-01-03

**Authors:** Paul Tewson, Mara Westenberg, Yongxin Zhao, Robert E. Campbell, Anne Marie Quinn, Thomas E. Hughes

The graph shown in Figure 3C is mislabeled. The drug that followed the ionomycin was PDBu (not carbachol). The description in the figure legend is correct. 

